# Reply to: Mitochondrial dysfunction as the missing link between circadian syndrome and dementia

**DOI:** 10.1016/j.tjpad.2025.100126

**Published:** 2025-03-10

**Authors:** Linling Yu, Xiong Wang

**Affiliations:** a Department of Laboratory Medicine, Tongji Hospital, Tongji Medical College, Huazhong University of Science and Technology, Wuhan 430030, China; b Department of Public health, Tongji Hospital, Tongji Medical College, Huazhong University of Science and Technology, Wuhan 430030, China

Dear Editor,

We are grateful for the opportunity to review the Letter to the Editor titled “Mitochondrial Dysfunction as the Missing Link between Circadian Syndrome and Dementia.” The authors present a compelling and insightful argument regarding the potential mechanistic role of mitochondrial dysfunction in bridging circadian syndrome (CircS) and neurodegenerative diseases. Their discussion builds upon our recent study, which provided epidemiological evidence linking CircS severity to an increased risk of dementia [1]. While their perspective highlights a critical mechanistic pathway that merits further exploration, we would like to offer a few comments and considerations for further discussion.

First, the authors appropriately emphasize the role of the suprachiasmatic nucleus (SCN) in modulating multiple endocrine axes, including the hypothalamic-pituitary-gonadal (HPG), hypothalamic-pituitary-adrenal (HPA), and hypothalamic-pituitary-thyroid (HPT) axes [2]. While hormonal dysregulation is indeed implicated in mitochondrial dysfunction[3], the direct causal relationship between these factors in the context of CircS remains to be fully elucidated. Additional experimental or clinical evidence demonstrating a direct mechanistic link between endocrine disruption and mitochondrial dysfunction in CircS could further strengthen this hypothesis.

Second, the discussion on mitochondrial oxidative phosphorylation (OXPHOS) and bioenergetic stress is highly relevant, particularly in the context of aging neurons and metabolic diseases[4]. It might also be valuable to consider other key mitochondrial processes, such as mitophagy, reactive oxygen species (ROS) homeostasis, and mitochondrial dynamics (fusion and fission), which are also critically involved in neurodegenerative disease progression[5]. These processes could provide additional mechanistic insights into how mitochondrial dysfunction mediates the link between CircS and dementia.

Furthermore, while the authors propose several potential therapeutic strategies, including circadian rhythm modulation, testosterone restoration, and metabolic interventions (e.g., time-restricted feeding and melatonin supplementation[6,7], the translational feasibility and efficacy of these approaches would benefit from further validation in human studies. Given the complex and multifaceted nature of CircS and its interactions with metabolic and neurodegenerative pathways, a more comprehensive therapeutic framework may be worth considering.

Overall, this Letter offers a thought-provoking perspective on the role of mitochondrial dysfunction in CircS-related neurodegeneration. We commend the authors for their efforts in advancing this important discussion and encourage further empirical studies to explore the proposed mechanisms. We thank the journal for the opportunity to engage in this dialogue and look forward to continued research in this promising area.

Yours sincerely,

## Declaration of competing interest

None
